# Experiments on the Electrical Conductivity of PEG 400 Nanocolloids Enhanced with Two Oxide Nanoparticles

**DOI:** 10.3390/nano13091555

**Published:** 2023-05-05

**Authors:** Elena Ionela Chereches, Alina Adriana Minea

**Affiliations:** Faculty of Materials Science and Engineering, Technical University “Gheorghe Asachi” of Iasi, Bd. D. Mangeron No. 63, 700050 Iasi, Romania; elena-ionela.chereches@academic.tuiasi.ro

**Keywords:** PEG 400, MgO, TiO_2_, electric conductivity, nanocolloids

## Abstract

This paper aims to provide some insights into the pH and electrical conductivity of two classes of nanocolloids with PEG 400 as the base fluid. Thus, nanoparticles of two oxides—MgO and TiO_2_—were added to the base fluid in 5 mass concentrations in the range 0.25–2.5 %wt. The stability was evaluated in terms of pH at ambient temperature, while the electrical conductivity was discussed at both ambient temperature and up to 333.15 K. The electrical conductivity of PEG 400 was previously discussed by this group, while the behavior of the new nanocolloids was debated in terms of the state of the art. More precisely, the influence of MgO increases electrical conductivity, and an enhancement of up to 48% for 0.25% MgO was found, while the influence of TiO_2_ nanoparticles was found to be in similar ranges. In conclusion, electrical conductivity varies with temperature and the addition of nanoparticles to the base fluid, although the mechanisms that are driving the nanoparticle type and concentration influence are not yet entirely assumed in the available literature.

## 1. Introduction

Nanocolloids receive increasing amounts of attention each year, and this is obvious from scrutinizing the most relevant repositories. Nanocolloids are suspensions of different nanoparticles within a number of base fluids, and the most popular ones are water or ethylene glycol (EG). In regard to nanoparticles, extremely various types are selected, such as, for example, carbon-based materials, silver, oxides, metals, or composites. Even if pioneering research was performed on alumina nanoparticles (NPs), special attention has lately been given to cheap and environmentally friendly NPs, such as, for example, MgO and TiO_2_ [1,2]. Nevertheless, the majority of research is focused on thermal conductivity and viscosity, and extremely little information can be found in regard to electrical conductivity or pH. Even if discussing MgO nanoparticles suspended in different base fluids, no study was found in connection with their electrical properties. Alternatively, TiO_2_ NPs were studied in combination with water, EG, or water + EG in terms of electrical conductivity variation at ambient temperature or when heated [3,4,5,6,7,8,9]. For example, Angayarkanni and Philip [3] studied nanofluids (NFs) with 13.5 nm of TiO_2_ in water and perceived an enhancement of 8876% in electrical conductivity at ambient temperature for a 4 %vol. concentration of NPs. Wang and Zou [10] performed an interesting study on functionalized TiO_2_ nanoparticles (β-CD/TiO_2_) inserted into oil-based drilling fluids. The manufactured new fluids were investigated in terms of thermal conductivity, and a clear advantage was found in terms of the enhancement of thermal conductivity. Another application of TiO_2_-nanoparticle-enhanced fluids was discussed by Wang et al. [11]. More precisely, authors studied the thermal conductivity of modified TiO_2_ nanoparticles via supramolecular β-CD suspended in a mixture of water and EG for the purpose of cooling data centers. Shoghl et al. [12] discussed the electrical conductivity of different nanofluids, including TiO_2_ dispersed in water with the addition of a surfactant, and concluded that the surfactant influences the values of electrical conductivity.

Lower TiO_2_ concentrations were adopted for other authors (i.e., max. 0.5 %vol.) [5,6,7], and an upsurge in electrical conductivity of almost 77% was reported for new fluids based on water + EG.

The electrical conductivity and pH of MgO nanofluids with EG as the base fluid were discussed by Mehrabi et al. [13] in terms of a polynomial neural network algorithm using several experimental datasets. The authors’ conclusion was that the application of an adaptive neuro-fuzzy inference system and genetic algorithm polynomial neural network approaches as a function of concentration, temperature, and NPs’ size provide accurate results. The datasets were collected from Adio et al. [14], who debated the factors that influenced the electrical conductivity and pH of MgO–EG NFs. Adio et al. [14] concluded that the upsurge in MgO concentration leads to the clear enhancement of EC and solution pH, and noticed no major influence of the sonication process on experimental values. The results of the pH of the nanofluids with MgO were situated in the region of 9.66–10.84, values about 50% higher than those of a base fluid (i.e., ethylene glycol). Additionally, a major augmentation was noticed in terms of electrical conductivity at room temperature, while the explanation relied on the electric double layer thickening due to the low ionic strength of the nanofluid samples. A similar explanation of this phenomenon was also offered by Posner [15], who discussed the properties and the electrokinetic behaviour of non-dilute colloidal suspensions.

Adio et al. [16] manufactured suspensions between MgO and EG with nanoparticle sizes of 20 and 40 nm, after which they measured the electrical conductivity with CON 700 equipment (EUTECH Instruments, Vernon Hills, IL, USA) between 293.15 and 363.15 K. The authors’ results revealed an increase in EC with an increase in temperature as well as differences depending on the size of NPs.

As mentioned previously, the adoption of polyethylene glycol (PEG) as a base heat transfer fluid is limited, with few studies in the available literature [17], while its electric behavior cannot be described with theoretical models [17,18,19,20,21,22,23,24,25,26,27,28,29,30].

Minea [31] discussed a number of phase change materials, along with PEGs of different molar masses with nanoparticles. The principal aim of their analysis was to highlight the impact of adding NPs (nanoparticles) on most of the relevant properties of the discussed suspensions. With this in mind, the experiments performed by the authors demonstrated a reasonable upsurge in viscosity, although thermal conductivity and specific heat were also enhanced.

Chereches et al. [30] suspended MWCNT nanoparticles in PEG 400 and noticed the influence of NPs on suspensions’ flow and electrical conductivity. More precisely, in terms of electrical conductivity, a consistent upsurge with both temperature and the addition of NPs was detected. The authors further declared that this is the usual comportment owing to the nature of MWCNTs.

For example, Awin et al. [32] reported the electrical conductivity of a number of lowly concentrated nanofluids with titanium dioxide dispersed in pure water. They concluded that an escalation in electrical conductivity (EC) appears when the concentration of NPs is higher. Similar outcomes were attained by other authors, such as, for instance Fal et al. [26], Zyla et al. [27], and Chereches and Minea [33]. The electrical conductivity of TiO_2_ dispersed in pure water was also reported by Sikdar et al. [4], who performed tests at four temperatures (i.e., up to 318 K) and noticed an upsurge of 80 times in electrical conductivity for the suspension with 3 %vol.

Even if the results are scattered, most of the authors proposed experimental correlations, while also noticing that the theoretical ones (see [18,19,20]) do not fit well with the experimental datasets. For example, several polynomial models were proposed by Chereches and Minea [32], as were logarithmic ones (see Islam et al. [5,6,7]), and power equations were proposed by Awin et al. [33] in addition to Fal et al. [29].

Even if EC (electrical conductivity) is an extremely less deliberated factor, a number of equations and models are discussed, compared, and evaluated in the current state of the art by a number of authors (see Chereches et al. [17] for more details). The classic models consider the concentration, shape, and size of NPs; however, none of them are capable of accurately predicting the experimental values. Some theoretical models are proposed by Maxwell [18], Bruggeman [19], and Fricke [20], mostly based on similarities with thermal conductivity, but fewer experimental models were discussed by research groups working on nanofluids research, and all are limited to a specific class of fluids (see, for example, water-based nanofluids or ethylene-glycol-based nanofluids) [17].

This paper’s aim is to shed some light onto PEG-400-based nanocolloids with two kinds of nanoparticles, MgO and TiO_2_, and it is a part of an extensive research project aiming to shed some light on PEG-based nanofluids enriched with oxide and MWCNT nanoparticles. The samples were prepared in a variety of mass concentrations, from 0.25 to 2.5 %wt., of oxide NPs. The results will be discussed in relation to suspensions’ stability (i.e., with the help of pH) and electrical conductivity of 293.15 K up to 333.15 K.

## 2. Chemicals and Experimental Procedure

The compounds for this study were from Sigma-Aldrich (St. Louis, MO, USA): PEG 400 (Kollisolv^®^ PEG E 400, CAS number 25322-68-3), TiO_2_ (CAS number: 13463-67-7), and MgO nanoparticles (CAS number: 1309-48-4). Nanofluids with PEG 400 were manufactured using a two-step method: the nanoparticles were dispersed into PEG 400 followed by 60 min of sonication to secure satisfactory stability and minimize the sedimentation of the naoparticles. For sonication, an ultrasonic bath, a Geti GUC02A (i.e., ultrasound power: 60 W, frequency: 40 Hz), was employed. Concentrations were carefully calculated to reach the anticipated nanocolloids with 0.25–2.5 %wt. concentrations (i.e., each sample concentration was calculated based on a mass fraction and 50 mL of each nanocolloid prepared based on the calculated quantities of PEG 400 and NPs). For weighing chemicals, an ENTRIS224l-1S balance from Sartorius AG (Goettingen, Germany,) with a precision of 0.1 mg, was employed.

Furthermore, the stability was evaluated based on both pH and electrical conductivity, as are outlined in the available literature [21,22,23,24,25] as possible methods with which to check the absence of sedimentation.

An Edge^®^ Multiparameter HI 2030 (Hanna Instruments, Woonsocket, RI, USA) with an incorporated temperature-measuring device possessing the capacity to perform wide measurements of electrical conductivity, up to resolutions of 500 mS/cm and 0.01 μS/cm resolution, was employed to determine electrical conductivity; a special sensor was utilized to determine the solution pH (i.e., HI-11310 sensor). The equipment can perform measurements in the temperature interval of 293.15–333.15 K with an uncertainty of ±0.2 K.

Measurements were performed at room temperature (i.e., 293.15 K) for pH as well as with variations in temperature for electrical conductivity. After each measurement set, a calibration operation was performed with the help of special calibration salts specific to the Edge^®^ Multiparameter HI 2030. Each measurement was repeated several times (i.e., 3–5 times) in order to avoid any uncertainty, and an average value was recorded for discussion of the experimental data.

The PEG 400 structure was analyzed previously via NMR spectroscopy and revealed a multiplet at 4.58 ppm corresponding to the proton of the OH as well as the presence of small quantities of water (more information can be found in Chereches et al. [30]). Additionally, Chereches et al. [30] discussed the results of Fourier transform infrared spectroscopy, differential scanning calorimetry, and thermogravimetric analysis applied to the PEG 400. The small traces of water existing in PEG 400 were also recognized via infrared spectroscopy.

All of the tests were performed at ambient pressure, and the global uncertainty of the experimental data was situated around 2.8%, which lies in an acceptable range for an experimental study.

## 3. Results and Discussion

When discussing new heat transfer fluids enhanced with nanoparticles, one of the most relevant aspects is connected to the stability of the suspensions. The sedimentation phenomenon occurs for most of the nanocolloids and has to be properly evaluated since it influences both the estimation of thermophysical properties as well as behavior in real-life applications. The next subsections will present the results of pH suspensions and the study of electrical conductivity. More precisely, the investigation of pH was directed towards suspensions’ stability, while the electrical conductivity experiment and the discussion of its results are useful for drawing attention to several possible applications of the investigated nanocolloids.

### 3.1. pH

**Figure 1:**
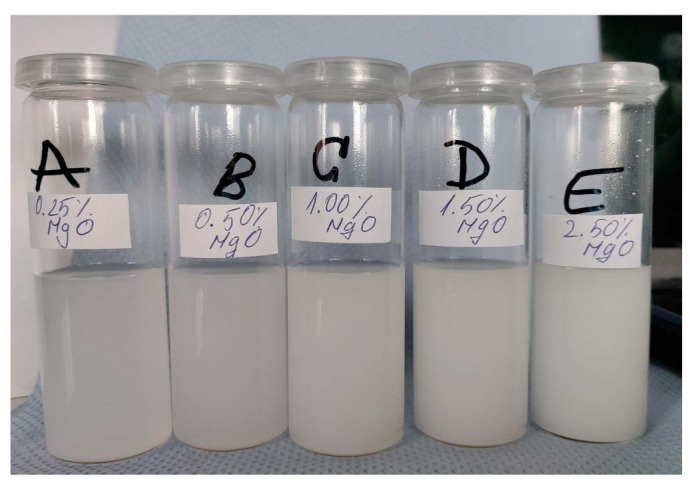
Samples: MgO nanocolloids with PEG 400.

**Figure 2:**
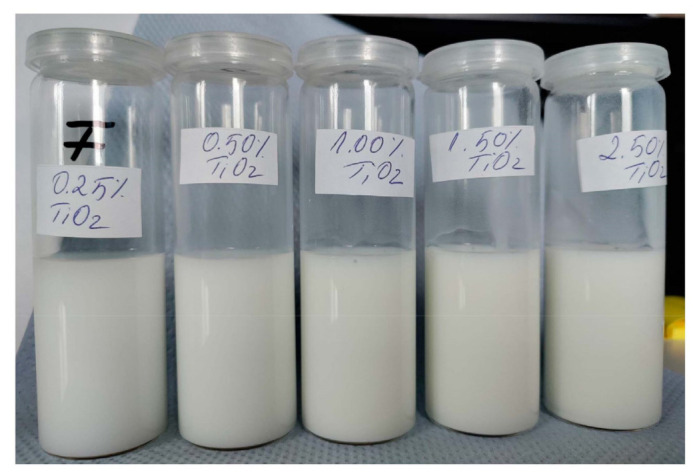
Samples: TiO_2_ nanocolloids with PEG 400.

The suspensions were tracked with a visual control (see Figure 1 for PEG 400 nanocolloids with MgO and Figure 2 for samples with TiO_2_) and pH for a period of 3 weeks, and no sedimentation was noticed. In terms of pH, it is clear from the available literature that it can indicate the stability of nanoparticle-enhanced fluids [21,22,23,24,25].

**Figure 3:**
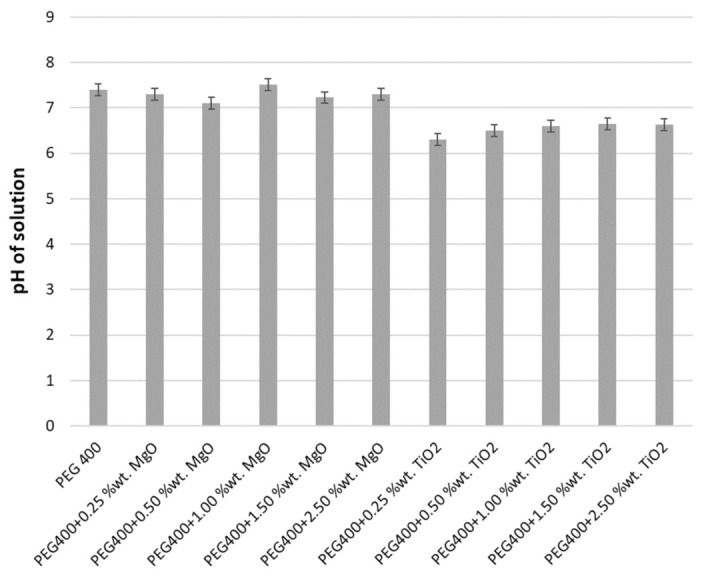
pH for PEG 400 and PEG-400-based nanocolloids at 293.15 K.

Additionally, Liu et al. [25] discussed this kind of approach in terms of a comparison between a zeta potential analysis (i.e., indicating the suspensions’ stability) and properties such as pH and EC, and concluded that the most stable nanocolloids are electrically balanced in addition to the fact that those with a low zeta potential are predisposed toward flocculation (i.e., clogs tend to be created). The samples’ pH was investigated at 293.15 K, after which experimental results were plotted (as shown in Figure 3). Figure 3 clearly indicates that all nanocolloids have a pH between 7.5 and 8.9. The measured pH values are in line with similar outcomes from the state-of-the-art literature, wherever pH = 7 is suggested for nanocolloids (see, for example, [21,22,23,24,25]).

Furthermore, from Figure 3 it can be seen that the pH is lower for TiO_2_ + PEG 400 nanocolloids if compared with the ones with MgO (i.e., the lowest value is about 15% lower than the PEG 400 pH).

If one compares the data with similar studies from the literature, they can consider the experiment performed by Adio et al. [14], who attained values between 9.66 and 10.84 for MgO dispersed in ethylene glycol of volume fractions up to 3%. These values are considerably lower than those obtained for the same type of nanoparticles dispersed in EG. Additionally, the data from Adio et al. [14] revealed that the addition of nanoparticles increases the pH of the solution, and this can be due to the occurrence of sedimentation. In any case, more experimental outcomes are required in order to be able to draw a solid conclusion on the significance of pH for the evaluation of stability (please see the results published by different authors, such as, for example, the complex study performed by Liu et al. [25]).

### 3.2. Electrical Conductivity Results

**Figure 4:**
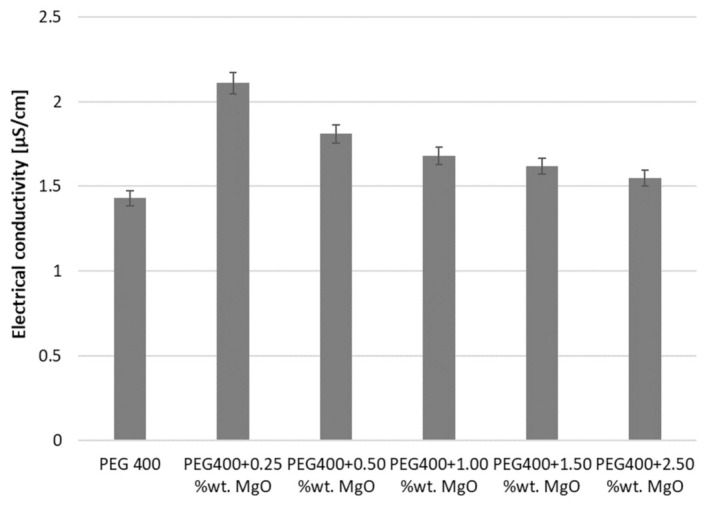
Experimental results at 293.15 K for PEG 400 + MgO.

As was affirmed in Section 2, two classes of nanocolloids were investigated and compared here in relation to their EC (electrical conductivity): First, the results for MgO + PEG 400 will be discussed in regard to the effect of the addition of nanoparticles (see Figure 4) and temperature variation. The results for PEG 400 were previously determined (see [17] for further insights and comparison with the state of the art) and were used here as a basis for comparison.

As can be noticed, the addition of NPs increases electrical conductivity, a phenomenon that is followed by a reduction when NPs are added to the host fluid (i.e., PEG 400). This phenomenon has not been completely elucidated; nevertheless, it was previously noticed and can be attributed to the occurrence and properties of the electric double layer. An in-depth study that also considers other host fluids may shed some light on the influence of the base fluid as well (i.e., PEG 400 in this particular situation).

Considering the experimental data, it is obvious that the electrical conductivity declines with the addition of MgO, and this phenomenon mostly occurs due to the low electrical conductivity of MgO. It must be underlined here that MgO nanoparticles are excellent electrical insulators and very good thermal conductors. Nevertheless, a disagreement was noticed with results the published by Mehrabi et al. [13] and Adio et al. [16], who noticed that the influence of MgO nanoparticles on ethylene glycol is huge; an enhancement of over 1000% was noticed. Overall, the current experimental data are considerably lower than those attained by other research groups [13,14,15,16]. As a possible explanation, the influence of the base fluid is acknowledged.

**Figure 5:**
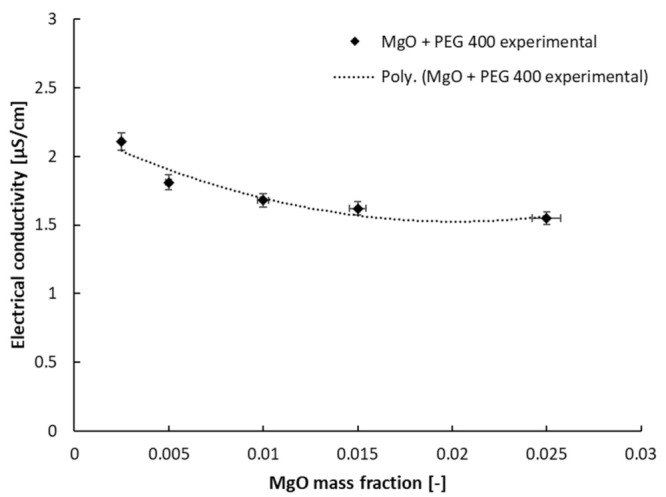
PEG 400 + MgO electrical conductivity variation with a mass fraction at 293.15 K.

If one analyses the experimental outcomes, portrayed in Figure 5, a decrease in electrical conductivity is clearly noticed; the decrease fits well on a second-degree polynomial trendline, as has also been demonstrated in other studies in connection with similar fluids (see [21,22,23,24,25]). Figure 5 illustrates the polynomial dependency of the electrical conductivity on the mass fraction, which can be estimated as follows:

σ = 1646.7 w^2^ − 66.449 w + 2.195; R^2^ = 0.92 (1)

where σ is the electrical conductivity and w refers to the mass fraction of MgO nanoparticles in the PEG 400. The R-squared value is satisfactory, and the equation can describe the influence of the addition of NPs on the σ values.

**Figure 6:**
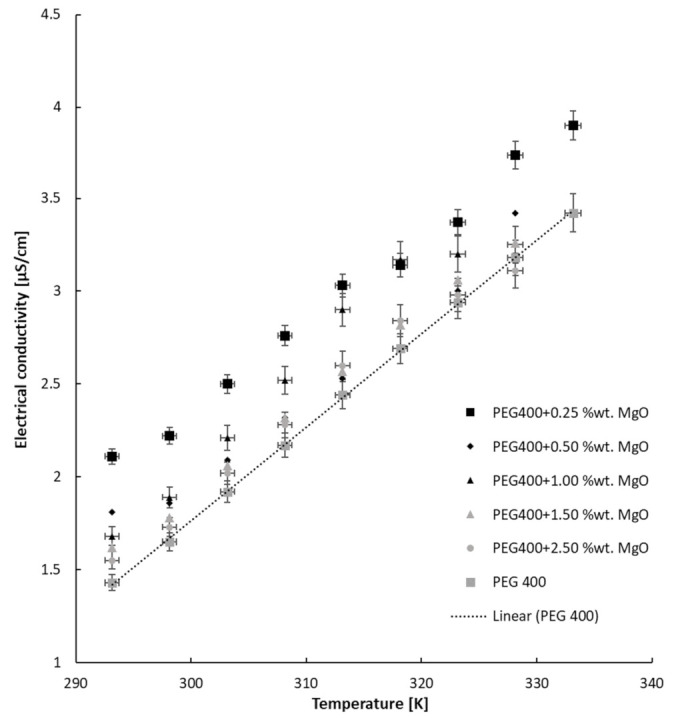
Nanocolloids’ electrical conductivity versus temperature.

The MgO nanocolloids’ EC deviation with temperature follows the PEG 400 variation, as is plotted in Figure 6. The experimental data are plotted in comparison with previously determined values for PEG 400, and one can see the linear amplification of electrical conductivity with temperature, following a linear equation:

σ = a T + b(2)

In Equation (2), parameters a and b are coefficients depending on each suspension, as are outlined in Table 1 for each fluid.

In conclusion, the enhancement of electrical conductivity with temperature is clear for all MgO nanocolloids and follows PEG 400 augmentation. To be precise, the values are approximately 2 times higher at 333.15 K for all fluids if compared with ambient temperature. The mechanisms of the increase in electrical conductivity are believed to be due to the electric double layer and conducting paths produced by the nanoparticles into PEG 400, as was also affirmed by Adio et al. [14], Fal et al. [26], and Zyla et al. [27]. In any case, the theoretical representations do not fit the experimental values, as was demonstrated by Minea [28].

**Figure 7:**
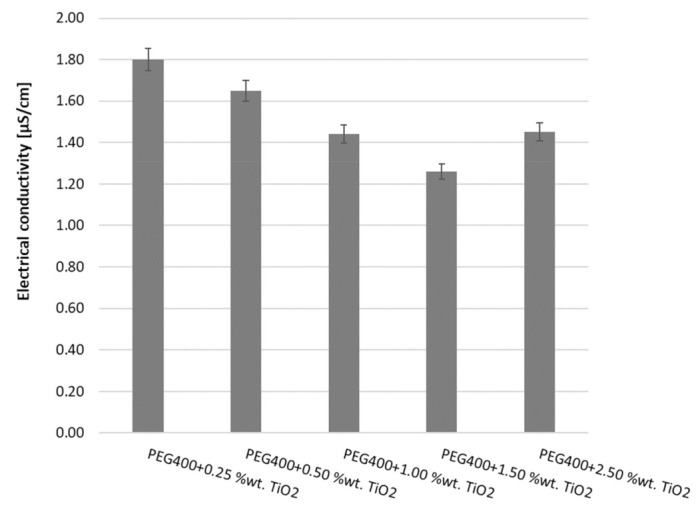
Experimental results at 293.15 K for PEG 400 + TiO_2_.

**Figure 8:**
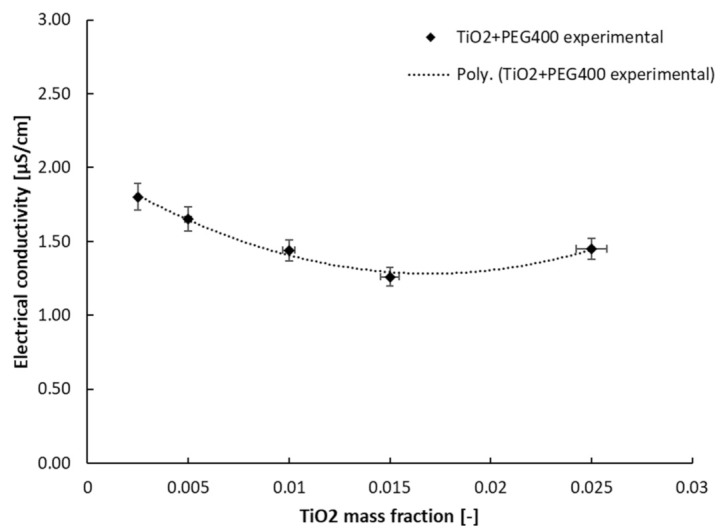
PEG 400 + TiO_2_ electrical conductivity variation with a mass fraction at 293.15 K.

**Figure 9:**
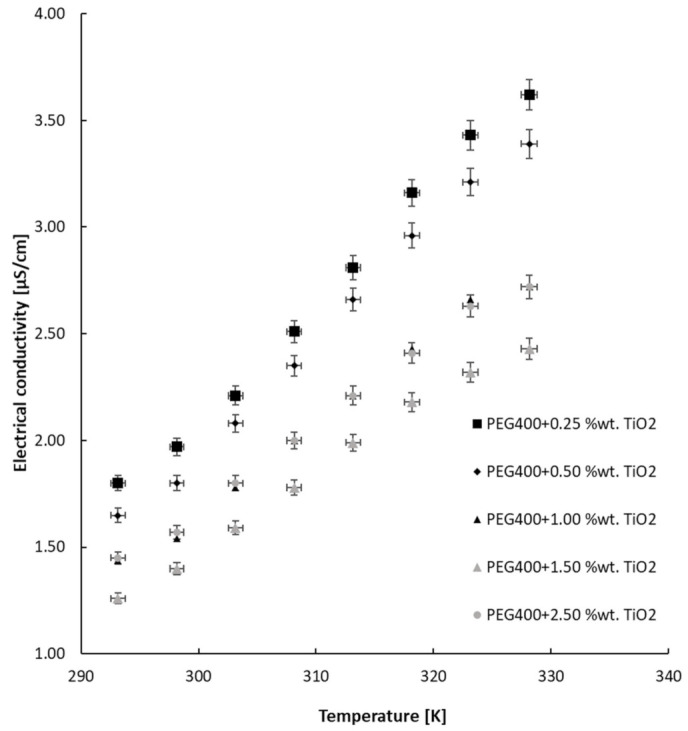
TiO_2_ nanocolloids’ electrical conductivity versus temperature.

Furthermore, the second class of nanocolloids’ experimental results will be discussed in terms of the variation in EC with a mass fraction at ambient temperature (see Figure 7 and Figure 8), as well as its variation with temperature (see Figure 9). The approach of the discussion of the results will be similar to the previous one.

As can be notice, the addition of NPs increases electrical conductivity, a phenomenon that is followed by a reduction in EC as the concentration of NPs increases. Subsequently, for large NP mass fractions the tendency of electrical conductivity is to increase. This phenomenon is due to TiO_2_ nanoparticles’ higher electrical conductivity.

Following the results obtained for the PEG 400 + MgO class of nanocolloids, Figure 8 displays the polynomial dependency of EC on the mass fraction, which can be estimated as follows:

σ = 2520.8 w^2^ − 85.703 w + 2.0109; R^2^ = 0.99 (3)

where σ is the electrical conductivity and w refers to the mass fraction of TiO_2_ nanoparticles in the PEG 400. The R-squared value is excellent, and the equation can describe the influence of the addition of NPs on the σ values.

The TiO_2_ nanocolloids’ electrical conductivity deviation depends on the samples’ temperature and follows the PEG 400 variation, as is plotted in Figure 9. The experimental data are plotted in comparison with previously determined values for PEG 400, and a linear increase in electrical conductivity with temperature can be seen, following an equation similar to Equation (2), where a and b are coefficients depending on each suspension, as outlined in Table 2 for each fluid with TiO_2_.

If one attempts a comparison with a similar nanofluid based on titanium oxide, they could use the data from Shoghl et al. [10], who demonstrated that electrical conductivity increases with the addition of NPs and is greatly impacted by the presence of a surfactant. The phenomenon identified by Shoghl et al. [10] for both an increase or decrease in electrical conductivity was also formation of the EDL or nanofluid stability.

If one follows the previously acknowledged models from the state of the art, as identified by Fal et al. [29], as well as with the help of a regression analysis performed by CurveExpert Pro: 2.7.3 software [34] for the two datasets, they can reasonably find the model type presented by Awin et al. [32]. The electrical conductivity of similar fluids (i.e., nanofluids with dispersed TiO_2_ nanoparticles) was discussed by Awin et al. [32], who proposed an exponential variation, which is also adopted here for both nanocolloids as follows:

(4) $$\frac{\sigma_{\mathrm{nf}}}{\sigma_{\mathrm{bf}}}=a{\left(\frac{T}{273.15}\right)}^{b}w_{}^{c}$$

where T is the temperature and w is the mass fraction, while a, b, and c are coefficients that have different values depending on nanocolloid type, as depicted in Table 3.

As a final remark, also taking into account the studies performed by this research group on PEG 400 with alumina and ZnO nanoparticles (see [17,32] for the results), electrical conductivity is decidedly subjective with regard to NP type, and its variation with temperature follows the tendency of the base fluid. In any case, the concentration of nanoparticles is also relevant, especially when discussing nanocolloids with MgO and TiO_2_. These authors believe that the mechanisms are more complicated and do not stand only in terms of the EDL phenomenon, as was affirmed in the available literature and discussed here. Nevertheless, it is clear that the oxide type and its intrinsic electrical conductivity substantially modify most base fluid properties. If one was to compare this study with previous research, they could assume that an experimental approach is the only solution for accurately estimating the electric behavior of each nanocolloid, since, at least for the moment, no similar behavior has been noticed and it is not possible to propose a universal model that can be valid for a class of NPs. Additionally, a comparison with a theoretical model was not considered useful in terms of this study conclusion, because most of the theoretical models only consider the volume fraction as a variation parameter, as was already demonstrated by Fal et al. [29] in a recent published study.

## 4. Conclusions

The electrical conductivity of two categories of oxide–PEG 400 nanocolloids was scrutinized. The nanocolloids were dispersed and manufactured using a two-step technique with mass concentration in the concentration array of 0.25 to 2.5 %wt. The experimental outcomes indicate an overall upsurge in electrical conductivity with the concentration of MgO and TiO_2_ NPs in PEG 400, which clearly hinges on the following features: nanoparticle structure and concentration. The most relevant augmentation was noted for suspensions with magnesium oxide, and it was around 48% for 0.25% MgO.

In any case, TiO_2_ + PEG 400 samples have relatively high growth in terms of electrical conductivity in similar experimental conditions. On the other hand, the dissimilarity of electrical conductivity with temperature was found to be linear for all nanocolloids, a phenomenon also noticed in PEG 400 variation.

Finally, it can be summarized that the addition of MgO nanoparticles is auspicious for the augmentation of electrical conductivity. Additionally, a few experimental models that were valid for each of the two investigated classes of nanocolloids were proposed.

The experimental outcomes were compared with similar results using other oxide nanoparticles, and no relevant similarities were noticed with regard to electrical conductivity values at ambient temperature.

Future Work

Nanocolloids with PEG 400 as the base fluid can be better choices for practical heat transfer applications at medium temperatures; one of the challenges is their stability.

Future work needs to be directed to thermophysical properties, and an analysis of the cost–benefit balance is of high relevance.

Thus, a coordinated approach to the study of liquid polyethylene glycol with different kinds of nanoparticles may deliver a good alternative to the market of new heat transfer fluids.

## Figures and Tables

**Table 1:** Coefficients connected to Equation (2) and statistical parameters.

Sample	a	b	R-Squared Value
PEG 400 + 0.25 %wt. MgO	0.0461	−11.472	0.99
PEG 400 + 0.50 %wt. MgO	0.0453	−11.594	0.97
PEG 400 + 1.00 %wt. MgO	0.0495	−12.782	0.95
PEG 400 + 1.50 %wt. MgO	0.0486	−12.66	0.99
PEG 400 + 2.50 %wt. MgO	0.0475	−12.367	0.98

**Table 2:** Coefficients connected to Equation (2) and statistical parameters.

Sample	a	b	R-Squared Value
PEG 400 + 0.25 %wt. TiO_2_	0.0524	−13.524	0.99
PEG 400 + 0.50 %wt. TiO_2_	0.0528	−13.893	0.99
PEG 400 + 1.00 %wt. TiO_2_	0.0398	−10.269	0.99
PEG 400 + 1.50 %wt. TiO_2_	0.0352	−9.0558	0.99
PEG 400 + 2.50 %wt. TiO_2_	0.0386	−9.9057	0.99

**Table 3:** Coefficients connected to Equation (4) and standard error.

Sample	a	b	c	Standard Error
PEG 400 + TiO_2_	0.55	−0.79	−0.14	0.06
PEG 400 + MgO	0.91	−1.21	−0.08	0.06

## Data Availability

Data can be made available on request.

## Nomenclature

R²	R-squared value, -
T	Temperature, K
w	Mass fraction, -
Greek Symbols	
φ	Volume fraction of nanoparticles, -
ϕ	Mass concentration of nanoparticles, %
ρ	Density, kg/m^3^
σ	Electrical conductivity, µS/cm
Subscripts	
bf	Denotes to base fluid
nf	Denotes to nanocolloid
p	Denotes to nanoparticles
Abbreviations	
EC	Electrical conductivity
EDL	Electrical double layer
EG	Ethylene glycol
NF	Nanofluids
NP	Nanoparticle
PEG	Polyethylene glycol
